# Four *Lentisphaerae* Family Metagenome-Assembled Genomes from the South Atlantic Ocean

**DOI:** 10.1128/mra.00496-22

**Published:** 2022-08-08

**Authors:** Oluwatayo A. Makinde, Percy Mutseka Lunga, Oliver K. I. Bezuidt, Thulani P. Makhalanyane

**Affiliations:** a Department of Biochemistry, Genetics and Microbiology, University of Pretoria, Pretoria, South Africa; University of Southern California

## Abstract

We present four *Lentisphaerae* metagenome-assembled genomes (MAGs) from the South Atlantic Ocean. The medium-quality genomes, affiliated with the family of *Lentisphaeraceae,* ranged from 4.86 to 5.46 Mbp and harbored the genetic capacity to produce secondary metabolites. This resource provides a basis for investigating the functional attributes of this phylum.

## ANNOUNCEMENT

*Lentisphaerae* is a member of the superphylum PVC (Planctomycetes, Verrucomicrobia, and Chlamydia) (1), known for their biotechnological importance and capacity to produce transparent exopolymers (2, 3). These constituents mediate the cycling of organic carbon particles between the euphotic zone and the deep ocean (4–7). Currently, there are only two validly described orders of this phylum, the *Victivallales* and the *Lentisphaerales*, due to difficulties associated with reproducing the ideal laboratory conditions required to isolate members of this species (7–10). Metagenome-assembled genomes (MAGs) have provided substantial insights regarding *Lentisphaerae* from several ecosystems (11–13). Nevertheless, we lack taxonomic and functional insights regarding *Lentisphaerae* from diverse ecosystems, such as the South Atlantic Ocean.

South Atlantic water samples (45 liters) were retrieved from three stations at a 5-m depth (Table 1), filtered through 0.2-μm polyethersulfone (PES) filter membranes (Merck, Republic of South Africa), and stored at −80°C until further processing. Metagenomic extractions were performed as described previously (14, 15) using the DNeasy PowerSoil kit (Qiagen, Hilden, Germany), and the resultant high-quality DNA was used to construct libraries using the KAPA HyperPrep kit (KAPA Biosystems, Massachusetts, USA) as detailed by the manufacturer. Sequencing was performed using an Illumina HiSeq 2000 platform (2 × 250 bp). Trimmomatic version 0.36 and PRINSEQ lite version 0.20.4 (-lc_method dust) were used to remove adaptors and overrepresented and low-quality reads from raw reads, respectively (16, 17). The resultant reads were assembled into contiguous segments using MegaHIT version 1.2.3 (18) and binned using MetaBAT 2 (Table 1) (19). CheckM version 1.0.18 was used to evaluate the completeness and contamination value of each MAG (20). The Genome Taxonomy Database Toolkit (GTDB-Tk) version 1.6.0 release 89 (P.-A. Chaumeil, A. J. Mussig, P. Hugenholtz, and D. H. Parks) was used to assign taxonomy, and the relative abundance of each MAG was determined using CoverM version 0.6.1 (21). Gene annotation was done using PGAP version 6.1 (22). The protein-encoding regions were identified using Prodigal version 2.6.3 (23), and the amino acid identities (AAI) of the four *Lentisphaerae* MAGs were calculated using AAI calculator (24) against the Lentisphaera araneosa genome sequence (GCF_0000170755.1), which had the highest similarly as determined using GTDB-Tk classification. The biosynthetic potentials of the four *Lentisphaerae* were determined using antiSMASH version 5.2 (25), using the strict detection mode option. The default parameters were used for all software unless otherwise indicated.

**TABLE 1:** Summary of genome statistics and comparisons of four novel *Lentisphaerae* MAGs, as well as sampling coordinates

Characteristic	Value or description for^*a*^:			
	Lentisphaeraceae_S22.bin.5	Lentisphaeraceae_S24.bin.22	Lentisphaeraceae_S14.bin.16	Lentisphaeraceae_S10.bin.24
Sampling coordinates	47°28.906′S, 09°59.970′W	47°28.906′S, 09°59.970′W	38°22.947′S, 11°14.942′W	36°35.591′S, 10°06.384′W
Depth of sequencing per metagenome (Gbp)	6	6	6	6
Bin coverage/relative abundance (%)	0.95	0.77	0.59	1.42
Genome size (bp)	5,465,546	5,228,557	4,867,689	5,264,327
G+C content (%)	39.34	39.03	40.84	39.19
Estimated genome completeness (%)	94.8	89.02	78.63	95.14
Estimated genome contamination (%)	9.42	4.17	8.16	6.93
Genome quality	Medium	Medium	Medium	Medium
No. of contigs	655	731	738	177
Largest contig (bp)	42,385	33,909	34,929	1,5864,44
*N*_50_ (bp)	10,158	8,194	7,627	44,916

No. of genes associated with:				
Protein coding	4,943	4,780	4,646	4,337
tRNA	53	35	36	38
rRNA	2	0	2	3
Secondary metabolic pathway for:				
Aryl polyene	1	2	2	0
Ripp-like	1	1	1	0
Ectoin	1	0	1	1
NRPS	0	0	1	0
Terpene	1	1	1	1

NCBI accession no. for:				
Raw reads	SRR15221251	SRR15221249	SRR15221260	SRR15221242
Assembly	JAJPOT000000000	JAJPOS000000000	JAJPOR000000000	JAJPOQ000000000
AAI with GCA_000170755.1_ASM17075v1 (%)^*b*^	48.5	48.58	47.95	49.38
Name of isolate with highest-similarity 16S rRNA gene	Uncultured bacterium clone	NA	NA	NA
GenBank accession no.	KF771565.1	NA	NA	NA
% similarity	98.98	NA	NA	NA

a NA, not available.

b AAI, amino acid identity.

The four *Lentisphaerae* genomes were classified as medium-quality draft genomes, consistent with criteria established by the Genomic Standards Consortium (26). These genomes were assigned to the *Lentisphaeraceae* family based on GTDB-Tk classification. The AAI ranged from 47.95 to 49.38%, based on the reference genome with the closest placement (L. araneosa). This result suggests that the four genomes belong to the same family as L. araneosa (GCF_0000170755.1) (Table 1). Interestingly, all the genomes harbored genes for unique secondary metabolic pathways, including terpene, aryl polyene, Ripp-like, ectoin, and nonribosomal peptide synthetase (NRPS) (Table 1).

### Data availability.

The NCBI BioProject accession number is PRJNA748242, while the Whole Genome Shotgun project has been deposited at DDBJ/ENA/GenBank and the accession numbers are given in Table 1.
